# Morphometric Study of Craters on Saturn’s Moon Rhea

**DOI:** 10.3847/psj/ac32d4

**Published:** 2021-11-25

**Authors:** Betzaida Aponte-Hernández, Edgard G. Rivera-Valentín, Michelle R. Kirchoff, Paul M. Schenk

**Affiliations:** 1 Lunar and Planetary Institute, Universities Space Research Association, Houston, TX 77058, USA;; 2 Southwest Research Institute, Boulder, CO 80302, USA

**Keywords:** Impact phenomena (779), Surface processes (2116), Saturnian satellites (1427)

## Abstract

Morphometric studies of impact craters on icy moons can be used to understand modification of crater topography. Several processes (e.g., viscous relaxation, ejecta deposition, repeated and overlapping impacts) act to shallow crater depth and relax the crater wall slope to similar or varying extents. Resolving these processes can help constrain the interior structure and surface properties of icy moons. Here, using morphometric measurements of craters on Rhea, we aim to constrain the processes that led to the observed crater population. We measured crater diameter, depth, and wall slope, as well as overall crater morphology (e.g., simple versus complex craters). Our results indicate that there exists a linear correlation between impact crater depth-to-diameter ratio and crater wall slope. This may suggest that the dominant modification process on Rhea is one that affects both properties simultaneously, which supports past heating events as the primary post-impact modification process. Additionally, the simple-to-complex crater transition for Rhea was found to be 12 ± 2 km, which is consistent with reported transition diameters for comparably sized icy bodies, indicating similar surface properties. A transition to shallower crater depths for large complex craters was not documented, indicating the absence of a rheological transition at depth in Rhea’s icy lithosphere, which may support the interpretation that Rhea is not fully differentiated.

## 1. Introduction

After an impact crater forms on an icy moon, its morphology over time can be modified by both endogenic (e.g., heating events, cryovolcanism) and exogenic processes (e.g., ejecta infill by cratering). In the case of Saturn’s moon Rhea, regional digital elevation models (DEMs) produced from Voyager and Cassini images show that there is no unambiguous record of cryovolcanic resurfacing due to the lack of observed plains (Moore & Schenk 2007). Comparison of crater shapes between Rhea and Iapetus, Saturn’s third-largest moon, showed that craters on Rhea have experienced topographic relaxation consistent with past heat flow events, while craters on Iapetus represent unrelaxed topographies (White et al. 2013). Indeed, thermal history calculations for Rhea indicate that subsolidus ice convection is possible (Ellsworth & Schubet 1983; Multhaup & Spohn 2007), which can contribute to the modification of its surface. (Multhaup & Spohn 2007) showed that convection may start around 330 Ma and take some 500 Ma to heat above the pure Ice I melting temperature. Such processes would alter the crater morphology by shallowing the crater depth while relaxing the crater wall slope. Additionally, although less dominant compared to Ganymede, Miranda, and Dione but still distinct, systems of normal faults cut across the surface of Rhea and in some places modify craters (Moore et al. 1985; Beddingfield et al. 2015).

Impact cratering is the dominant exogenic process to alter preexisting craters on Rhea, unlike on Saturn’s irregular satellite Helene, where E-ring deposition results in erasure of small craters on its leading hemisphere (Hirata et al. 2014). Repeated and overlapping impacts can infringe on crater topography, altering crater wall slopes. Ejecta deposition from nearby impacts can also shallow crater depths, particularly of smaller craters. This is important for surfaces that are at or near crater saturation equilibrium, which may be the case for some of the midsized moons (MSMs) of Saturn (Kirchoff & Schenk 2010; Kirchoff 2018). The final crater shape is also potentially a by-product of the stochastic impact cratering process. Differences in impactor characteristics, as well as differences in target properties, may affect the final observed crater morphology (Herrick & Hynek 2017).

Crater morphometry, the quantitative description of impact crater shapes, has been used to resolve the effects of exogenic and endogenic surface processes, as well as to compare planetary surfaces. Key measurements have included crater diameter (*D*) and depth (*d*) and the derived crater depth-to-diameter ratio (*d*/*D*). For Rhea, previous work determined average *d*/*D* values of 0.13 (Schenk 1989, 1991; Rivera-Valentín et al. 2012) and 0.221 (White et al. 2013) for small, simple craters. Other similar MSMs such as Iapetus and Dione have average *d*/*D* of 0.187 (White et al. 2013) and 0.105 (Schenk 1989) for simple craters, respectively. Values for icy moons appear to range from those collected from fresh, simple craters on rocky surfaces such as the Moon, where *d*/*D* ~ 0.2 (e.g., Pike 1974), to asteroidal-like values of *d*/*D* ~ 0.14 (Sullivan et al. 1996; Robinson et al. 2002; Burchell & Johnson 2005; Vincent et al. 2014), indicative of the importance of target mechanical properties.

Crater morphometry is also used to infer the simple-to-complex crater transition diameter (*D*_*t*_). This is the diameter where the crater morphology transitions from a simple, bowl-shaped crater to a complex crater with a flat floor and/or central peak. The transition crater diameter is largely a function of the body’s gravity and surface properties (Melosh 1989). For targets with similar surface properties, gravity scaling laws can be used to predict *D*_*t*_. Recently, Robbins et al. (2020) used a robust statistical treatment of Pluto’s and Charon’s crater morphometries to derive a *D*_*t*_ ~ 12 and ~15 km for Pluto and Charon, respectively. Owing to Charon’s and Rhea’s similar gravities, 0.29 m s^−2^ and 0.26 m s^−2^, respectively, a similar *D*_*t*_ is expected, assuming similar surface properties. However, the most recently suggested value for Rhea is *D*_*t*_ = 4.71 km (White et al. 2013), nearly a third of Charon’s, potentially hinting at vastly different surface properties.

Here we expand on previous work by collecting crater morphometry measurements on Rhea to study the dominant post-impact modification process regionally governing the surface of this icy satellite. Measurements were collected from a topographic map derived from photoclinometry, stereo, and shadow length measurements from Cassini high-resolution (0.18 km pixel^−1^) images (Schenk et al. 2011) collected by the Imaging Science Subsystem (ISS). We used multiple analytical techniques to study *d*/*D* and how it varies with crater morphology, as well as to derive *D*_*t*_ for Rhea. We also searched for other changes in crater morphology with size, as was found for Europa, Ganymede, and Callisto (Schenk 2002).

## 2. Crater Morphometry

### 2.1. Methods

**Figure 1.**
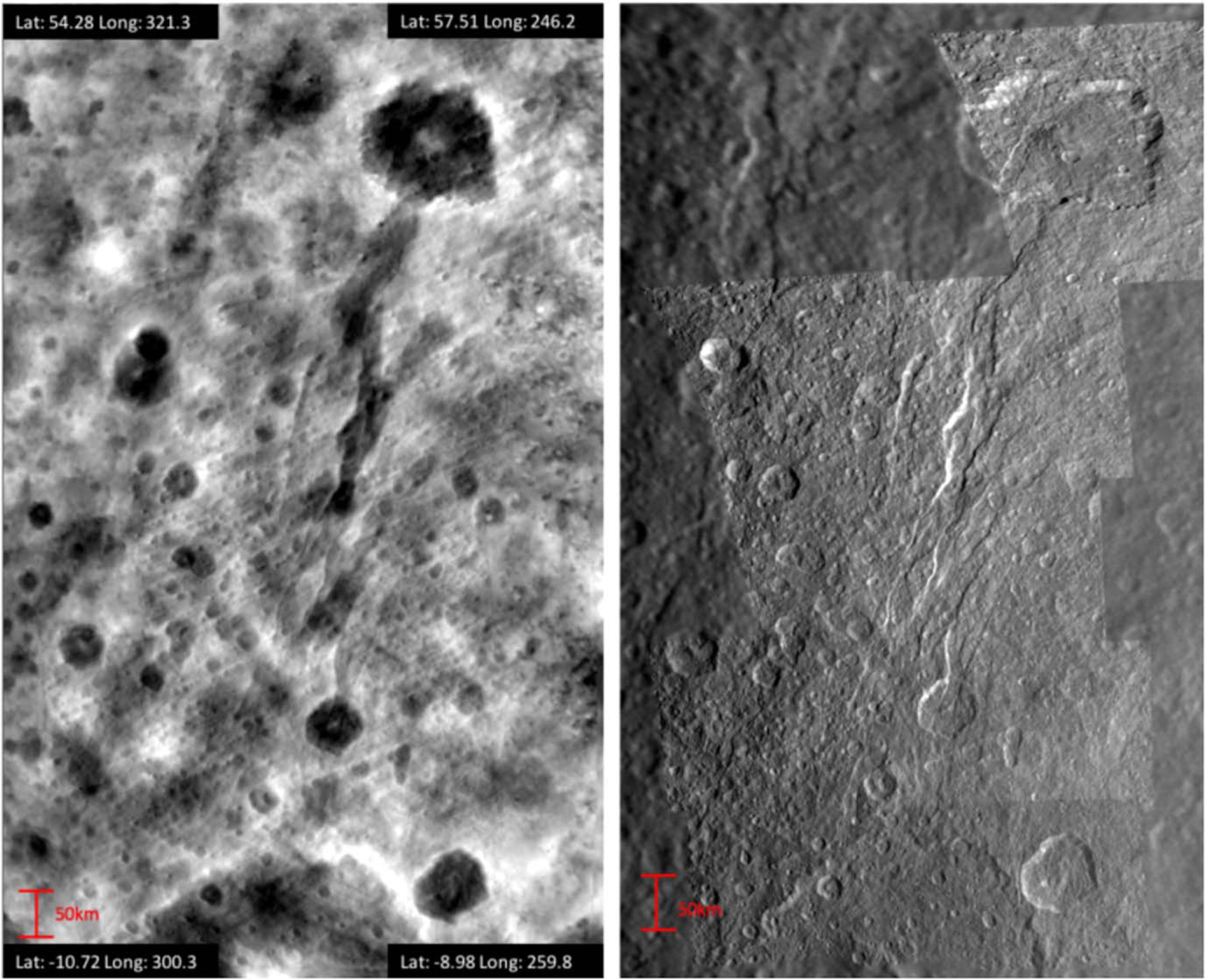
Topographic map used in our study (left) and the original image mosaic (right). For location reference, the biggest northern crater visible is Wakonda (*D* = 123 km), and in the southern part of the map you can see half of the Powehiwehi basin (*D* = 271.2 km). The map covers a ~475,000 km^2^ area in the trailing hemisphere of Rhea, extending over the equator, but it is mostly focused on the northern hemisphere. The center of the map corresponds to a latitude of 25° N and 280° W.

Measurements were collected from a regional topographic map derived from photoclinometry, stereo, and shadow length measurements from Cassini ISS high-resolution (0.18 km pixel^−1^) images (Schenk 2010; Schenk et al. 2011). Two methods were used in the process: stereo image analysis and two-dimensional photoclinometry of low-Sun regions. The latter allows the production of DEMs of larger surface areas, as Cassini stereo imaging is minimal (Schenk & Bulmer 1998; Schenk 2002; Schenk et al. 2011). The high-resolution topography map encompasses an area of ~475,000 km^2^ from 57°.5 N to 10°.72 S and from 246°.2 to 321°.3 W and thus is focused on the trailing hemisphere of Rhea. As seen in Figure 1, the location includes a generous amount of craters and Avaiki Chasmata, which cuts across the map from the Powehiwehi basin to Wakonda crater.

The resolution of the topographic map, 0.18 km pixel^−1^, limited crater identification. We applied a conservative 20-pixel limit (i.e., 3 km diameter craters), compared to the suggested minimum diameter limit of 10 pixels for crater counts (Wang et al. 2020), to assure robust crater identification. Because the smallest crater to be studied was 3 km in diameter and the simple-to-complex transition diameter for Rhea has been reported as *D*_*t*_ = 4.5 km (White et al. 2017) up to 15 km (Chapman & McKinnon 1986), we expected to sample both simple and complex crater morphologies.

**Figure 2.**
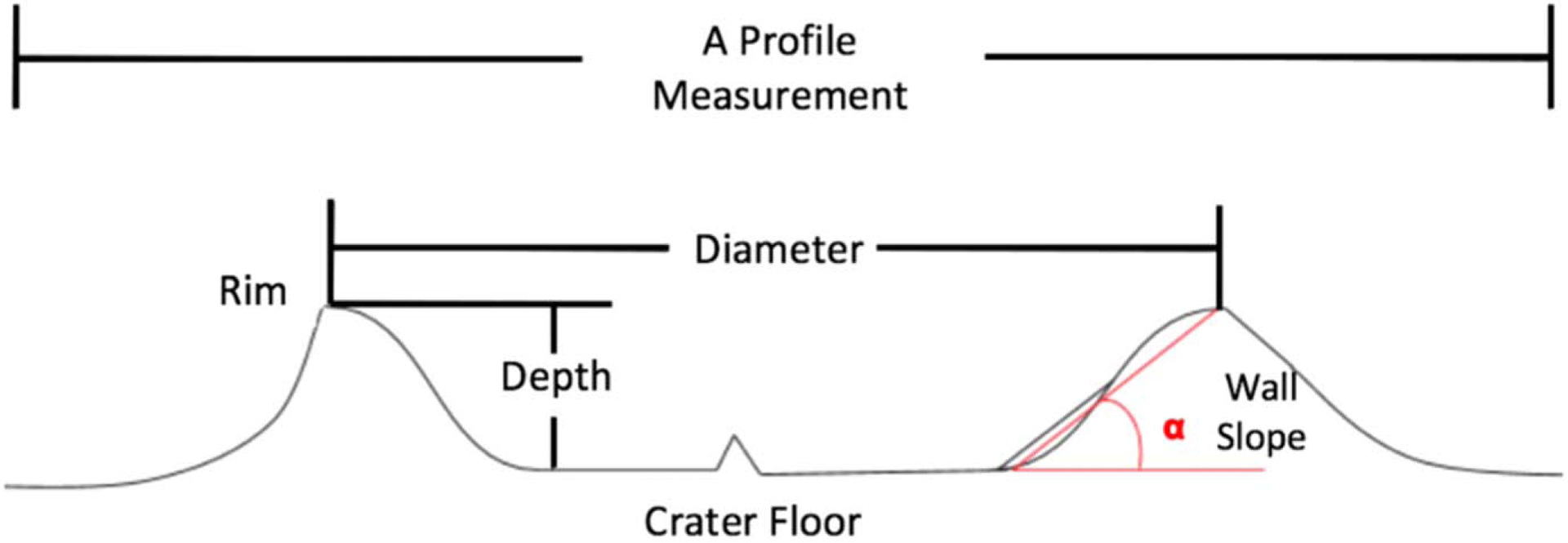
Illustration of the measurements taken for every identified crater.

The QVIEW tool in the Integrated Software for Imagers and Spectrometers v3 (ISIS3) software (Anderson et al. 2004) was used to measure the diameter (*D*) and depth (*d*) of each identified crater, as well as to assess the crater morphology. For every identified crater less than 80 km in diameter, crater topography was sampled via four topographic profiles that were separated by 45° in azimuth. For crater diameters greater than 80 km, two additional profiles were collected to ensure that the complexity of the depression was adequately sampled. In order to include the geologic context of the surrounding terrain, each profile was extended at least one crater radius in length beyond the crater rim. Each profile then provided two depth measurements (i.e., from the left rim to crater floor, and from the right rim to crater floor) and one diameter measurement. The final reported crater depth and diameter were then the average over the profile ensemble. The uncertainty of the measurement was considered as the sum in quadrature of the standard deviation of the profile ensemble and the topographic uncertainty of the map. Similarly, each profile provided two measurements of crater wall slope (*α*), which was calculated by finding the least-squares fit (LSF) slope (*β*) of the topography from the rim to the the crater floor and applying the geometric relation *α* = arctan (*b*). Uncertainty in *α* was found by propagating the uncertainty of the LSF fit through the averaging of the four profiles. In Figure 2, we illustrate the measurements that were taken.

**Figure 3.**
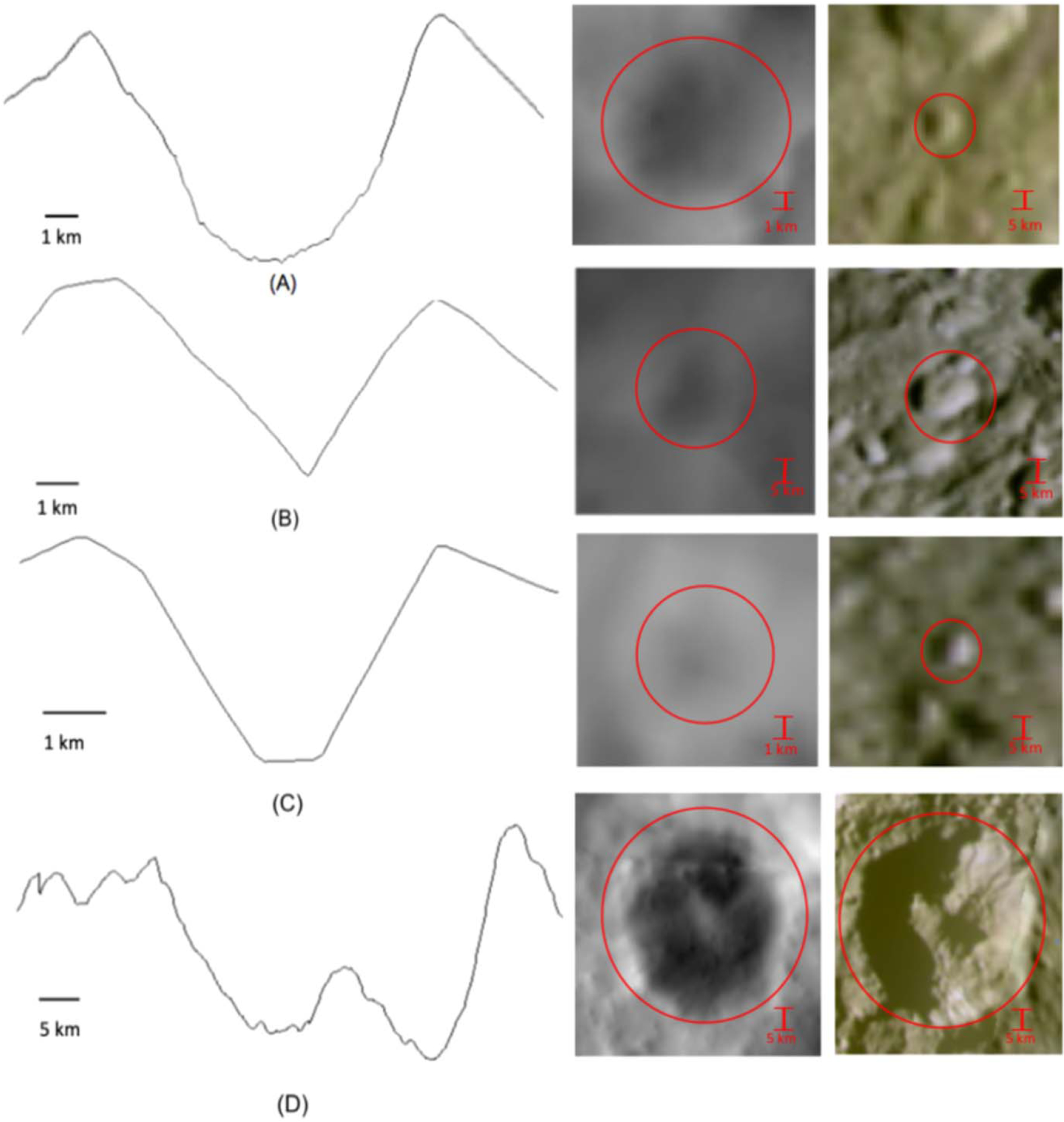
Examples of crater morphology classifications according to their physical features, with (a) a classical simple crater with a bowl shape, (b) a simple crater with a cone shape, (c) a flat-floor complex crater, and (d) a complex crater with a central peak. On the left we show an example topographic profile, in the middle we show the topography map centered on the crater, and on the right we show a Cassini ISS image of the crater. The red circle denotes the crater in both the topographic and ISS image.

For crater morphology, we categorized each topographic profile into either simple, complex, or ambiguous morphology. The assigned crater morphology was then the most often identified morphology from the ensemble of profiles for a given crater. Simple craters were defined by the classic bowl or cone shape, while complex craters were defined by a flat floor, including those often considered transitional between simple and complex and those with a central peak. An example crater for each morphology is shown in Figure 3. Note that since our largest diameter was limited to one *D* > 100 km, we did not see morphologies associated with basin-sized craters. Powehiwehi appears only partially in the studied region, and hence it was not included. Ambiguous morphologies were noted for those topographic profiles where insufficient detail was available to clearly distinguish between simple and complex morphology.

### 2.2. Results

We collected the diameter, depth, and crater wall slope of 742 craters from the topographic map in Figure 1; however, not all craters were included in our final analysis. Craters not included consisted of (1) craters with highly disrupted rims (e.g., rims superposed by at least one other crater, such as doublet craters), (2) craters partially out of our image range, and (3) ambiguous crater morphologies. Craters were studied regardless of their planform shape or degradation state. These filters resulted in a total of 509 craters for our analysis.

Crater diameters in the final data set ranged from 4.3 to 132 km. The slightly dominant morphology in the study area were complex craters at 51%. The largest simple crater identified was *D* ≈ 18 km, and the smallest complex crater identified was *D* ≈ 6 km. As such, there is significant overlap between these two crater morphologies with respect to crater diameter. On average, simple craters were characterized by *d*/*D* = 0.11 and crater wall slopes of 13°. Similarly, complex craters were characterized by *d*/*D* = 0.08 and crater wall slopes of 11°. As such, the *d*/*D* and crater wall slopes of these two crater morphologies alone were not two distinct groups in the overall population, which was inclusive of all degradation states. Results are summarized in Table 1.

**Figure 4.**
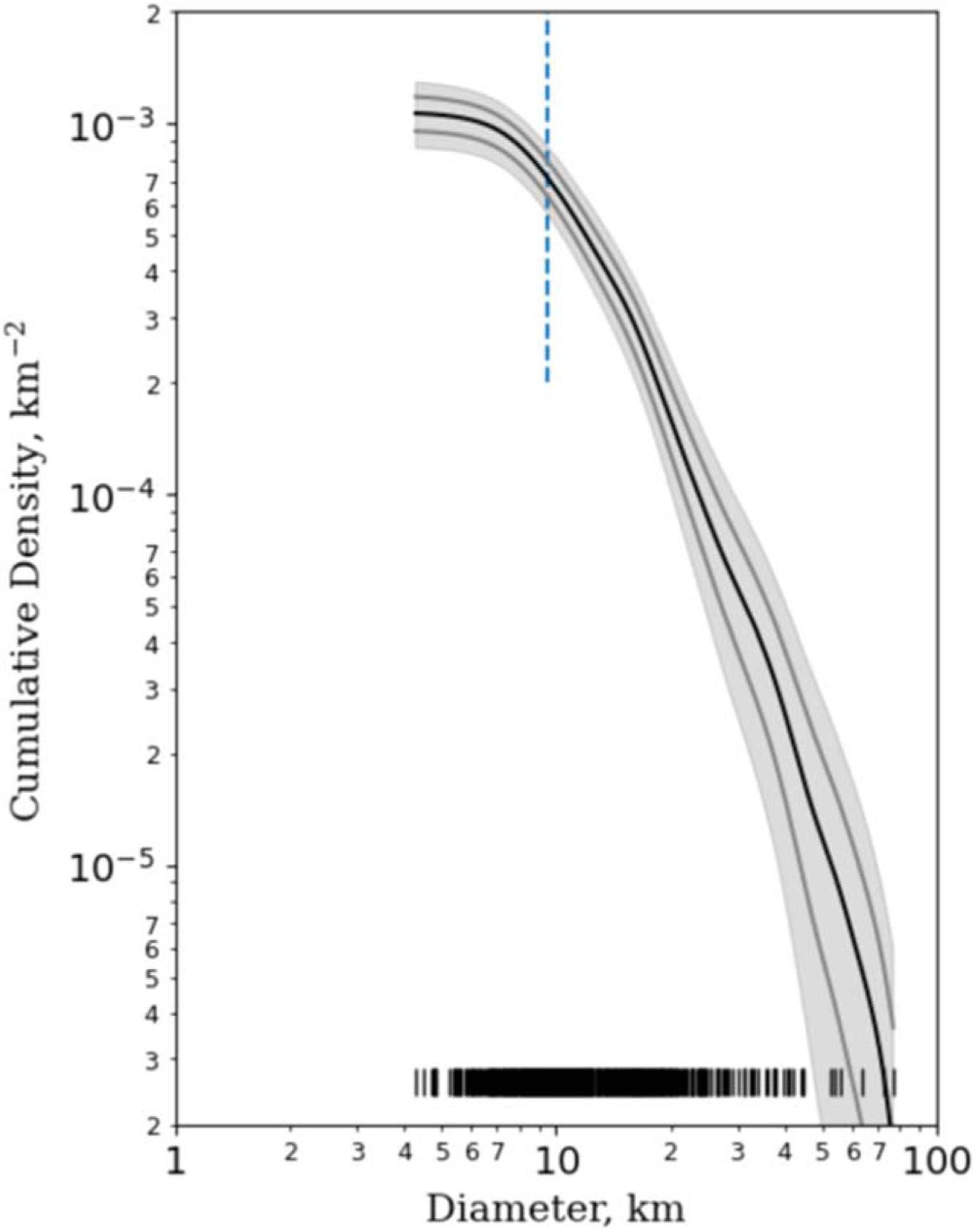
KDE version (Robbins et al. 2018) of the cumulative CSFD (black line). The gray envelope shows the 2*σ* uncertainties, while the gray line indicates the 1*σ* uncertainties, which are more similar to traditional Poisson error bars. The dashed blue line indicates the derived completeness limit for the crater counts based on where the differential SFD reaches its maximum multiplied by 10%. Small black lines at the bottom of the plot indicate each individual crater diameter measurement for reference.

To contextualize our study area, we studied the cumulative crater size–frequency distribution (CSFD) and compared with previous counts (Kirchoff & Schenk 2010), which accounted for some 35% of the surface. In Figure 4 our crater counts are shown using a kernel density estimate (KDE) version of the cumulative CSFD, which is a more robust statistical treatment of crater counts and is better suited to account for multiple sources of uncertainty (Robbins et al. 2018). This method plots unbinned data and sorts craters into a descending order by diameter. The KDE calculation assumes that each crater diameter measurement can be represented by a Gaussian, the center of the Gaussian is at the measurement, and the width is 10% of that measurement. The Gaussians for each measurement are then summed to create the SFD as seen in Figure 4. The uncertainty envelope is derived from the data with a Monte Carlo–style sampling, instead of being assumed to have a distribution (e.g., Poisson). Using the York method, which accounts for error in both the *x*- and *y*-direction (York et al. 2004), we found the best-fit power law to have a slope of −2.3 ± 0.9, which agrees well with previous crater counts by Kirchoff & Schenk (2010) that found that craters on Rhea followed a power law with a slope of −1.8.

**Figure 5.**
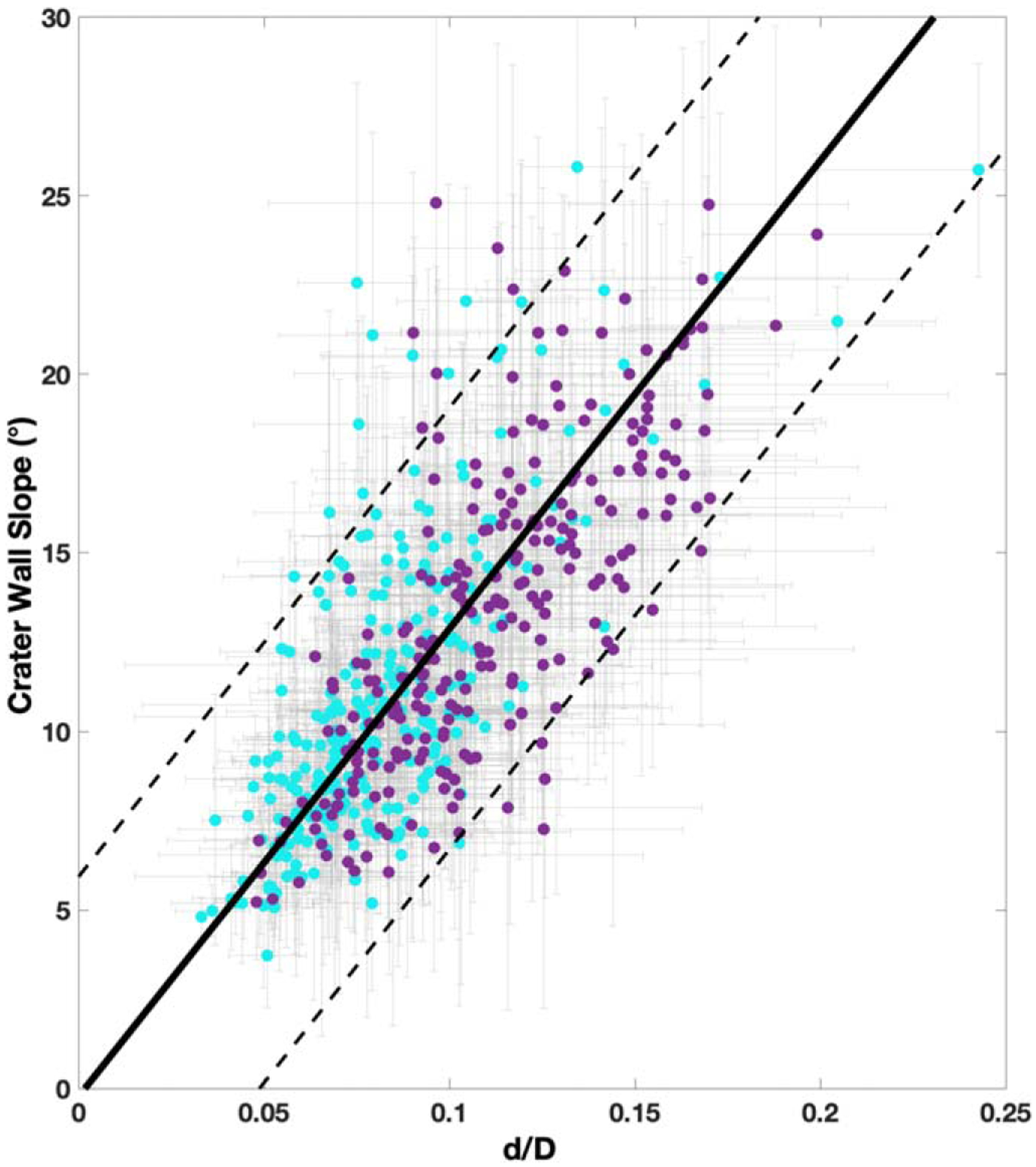
Depth-to-diameter ratio (*d*/*D*) vs. the crater wall slope, along with the linear fit in solid black for the total group of 509 craters. The gray lines show the uncertainty of each data point. The prediction interval to 95% confidence is shown by the dashed black lines. Crater morphology is shown in purple for the 248 simple craters and in cyan for the 261 complex craters.

To investigate the dominant post-impact crater modification process, we analyzed the relationship between crater wall slope and depth-to-diameter ratio (*d*/*D*), which is shown in Figure 5. In the overall population, *d*/*D* ranged from 0.03 to 0.24, with a mean value of 0.10 ± 0.03. The crater wall slopes ranged from 4° to 25°.8, with a mean value of 12°.3 ± 4°.4 (Table 1). The relationship between *d*/*D* and crater wall slope is well described by a linear fit following the York method (York et al. 2004), with a slope of 131 ± 9.6 and an intercept of −0.22 ± 0.8 (error to 95% confidence). Simple and complex craters are not distinct from the overall trend at a slope of 143 ± 18 and 149 ± 17, respectively. To identify craters that may warrant further analysis, in Figure 5 we include the prediction interval of the LSF to a 95% confidence. Here, for a given *d*/*D*, the prediction interval identifies predicted crater wall slope values for a 95% probability given the trend. We find that 4.9% (i.e., 25) of the craters studied fall outside of the LSF prediction interval and thus deviate from the identified trend.

**Figure 6.**
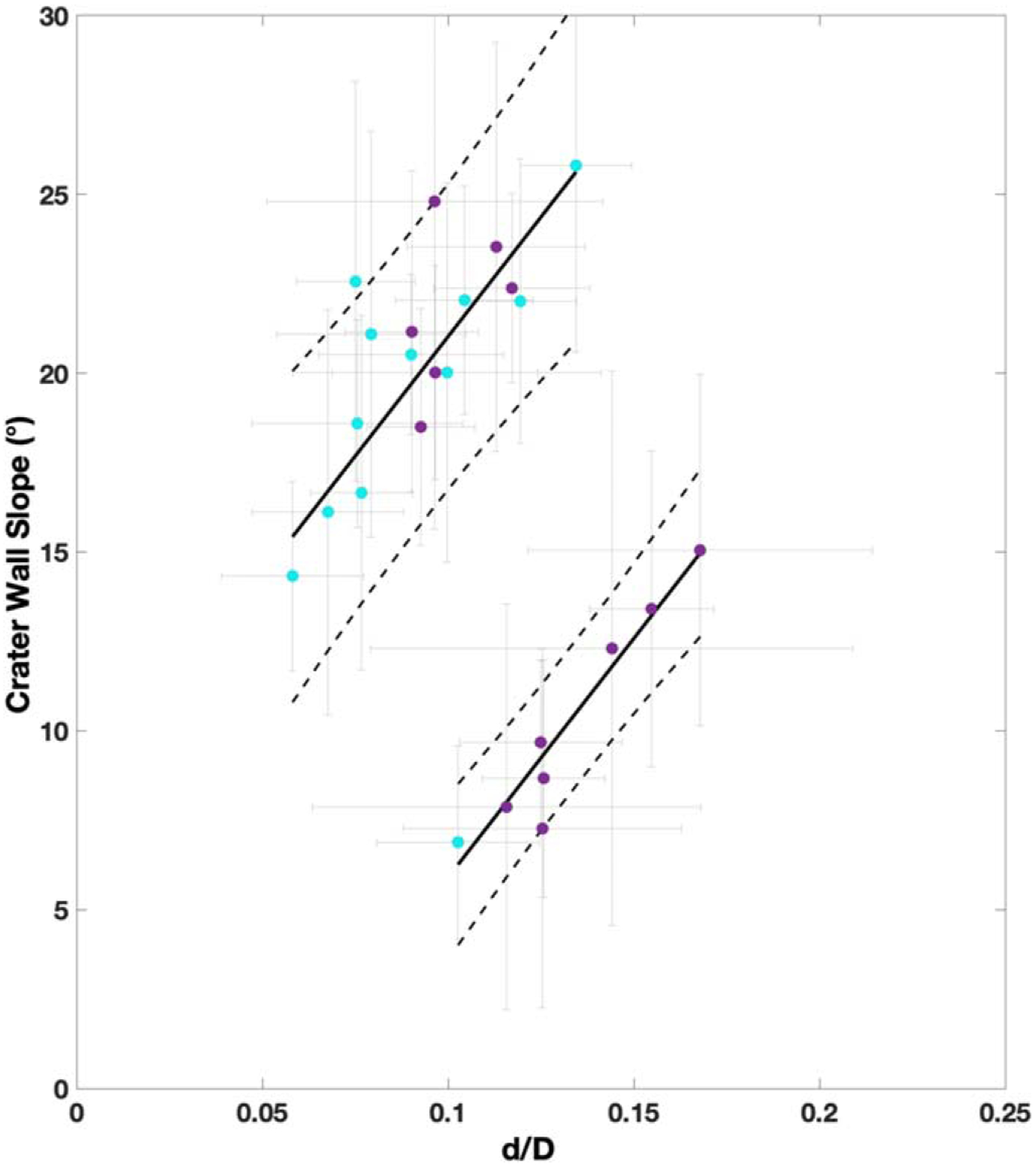
Depth-to-diameter (*d*/*D*) ratio vs. the crater wall slope for the two groups of craters outside of the prediction interval in Figure 5. The black solid line is the linear fit for each group, with the dashed black lines the prediction intervals to 95% confidence. The gray lines are the uncertainties.

In Figure 6, we further investigated the craters that fell outside of the LSF’s prediction interval of the overall population. These craters form two populations. The bottom set, so called because they fall below the 95% prediction interval in Figure 5, is characterized by shallow slopes, with an average wall slope of 10° ± 3°, and dominated by simple craters. On the other hand, the top set is characterized by steeper crater wall slopes, with an average of 21° ± 3°. The majority of these craters (~65%) are complex craters. Besides their morphometric differences, these two crater groups do not show any spatial relationship (e.g., clustering) in our study area that would suggest, for example, that they are possible secondaries. As such, they may represent part of the evolution of crater morphologies on Rhea.

## 3. Simple-to-complex Crater Transition Diameter

As discussed in Robbins et al. 2020, there are multiple ways of estimating simple-to-complex transition diameter (*D*_*t*_) in the literature, but no agreed-upon best practice as of yet that properly captures the uncertainty and the potential range of values. Thus, here we use four methods in order to robustly approximate this value.

**Figure 7.**
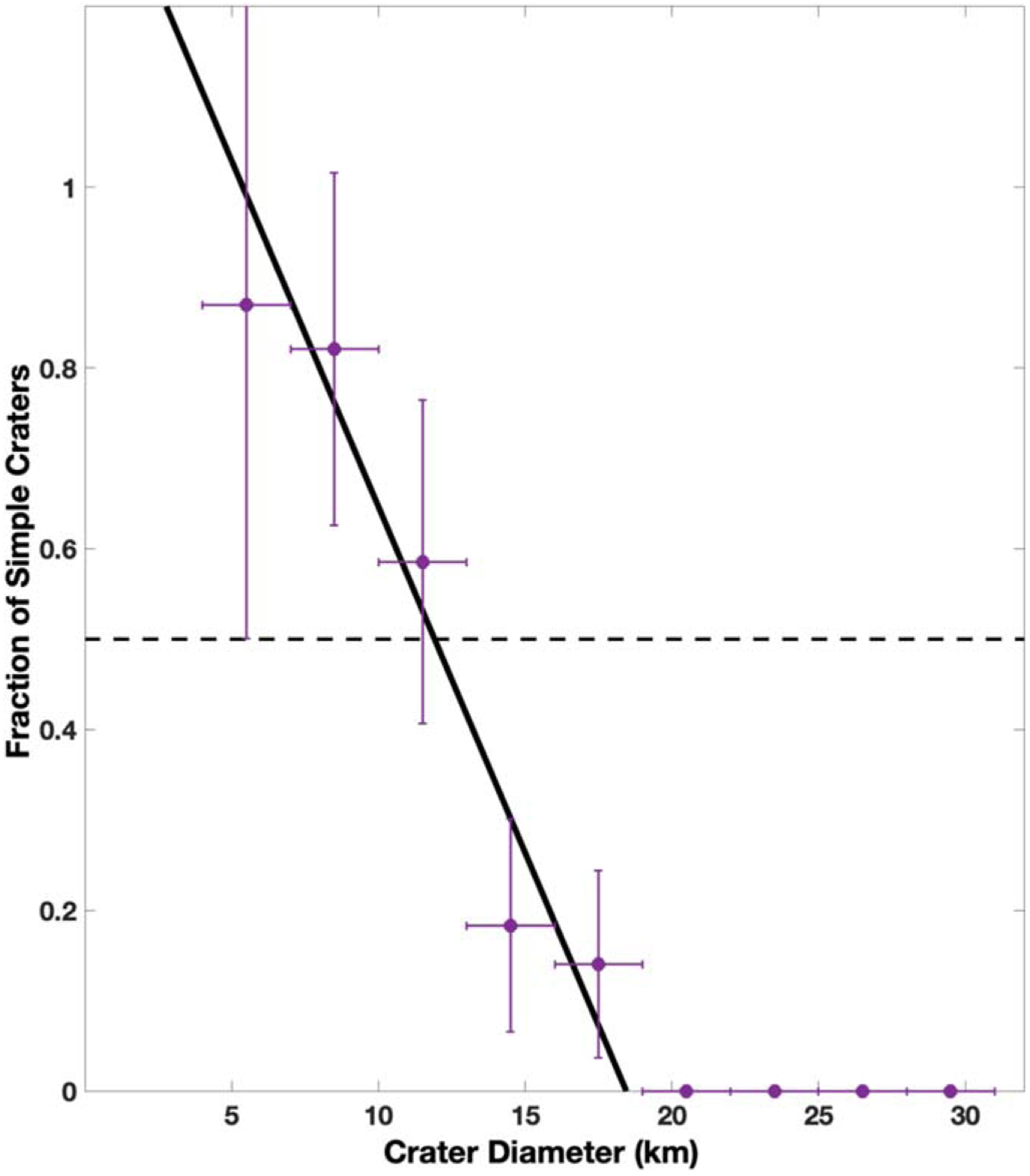
Fraction of simple craters as a function of diameter in bins of 3 km (purple circles). Bins greater than 35 km are omitted, as they continue the same trend of zero simple craters because the largest simple crater identified had a diameter of 18.1 km. The horizontal bar is the bin size, while the vertical bar is the propagated Poisson error of the crater counts in each bin. The black solid line is the LSF of the binned data. The dashed line marks where 50% of the craters are categorized as simple.

Pike (1980) suggested that the transition diameter was the diameter where 50% of craters had simple, bowl-shaped morphologies. As shown in Figure 7, we binned the crater population into 3 km bins and found the fraction of craters for each bin that were simple craters. The uncertainty in the fraction was found by assuming Poisson error for the crater counts and propagating through to the final fraction. The simple-to-complex transition diameter is then given as the diameter bin that intersects the 50% fraction, which occurs for the 11.5 km crater diameter bin. Additionally, we fit an LSF to the binned data to find the diameter that crosses the 50% simple crater fraction. This suggests a value of 11 ± 1 km.

**Figure 8.**
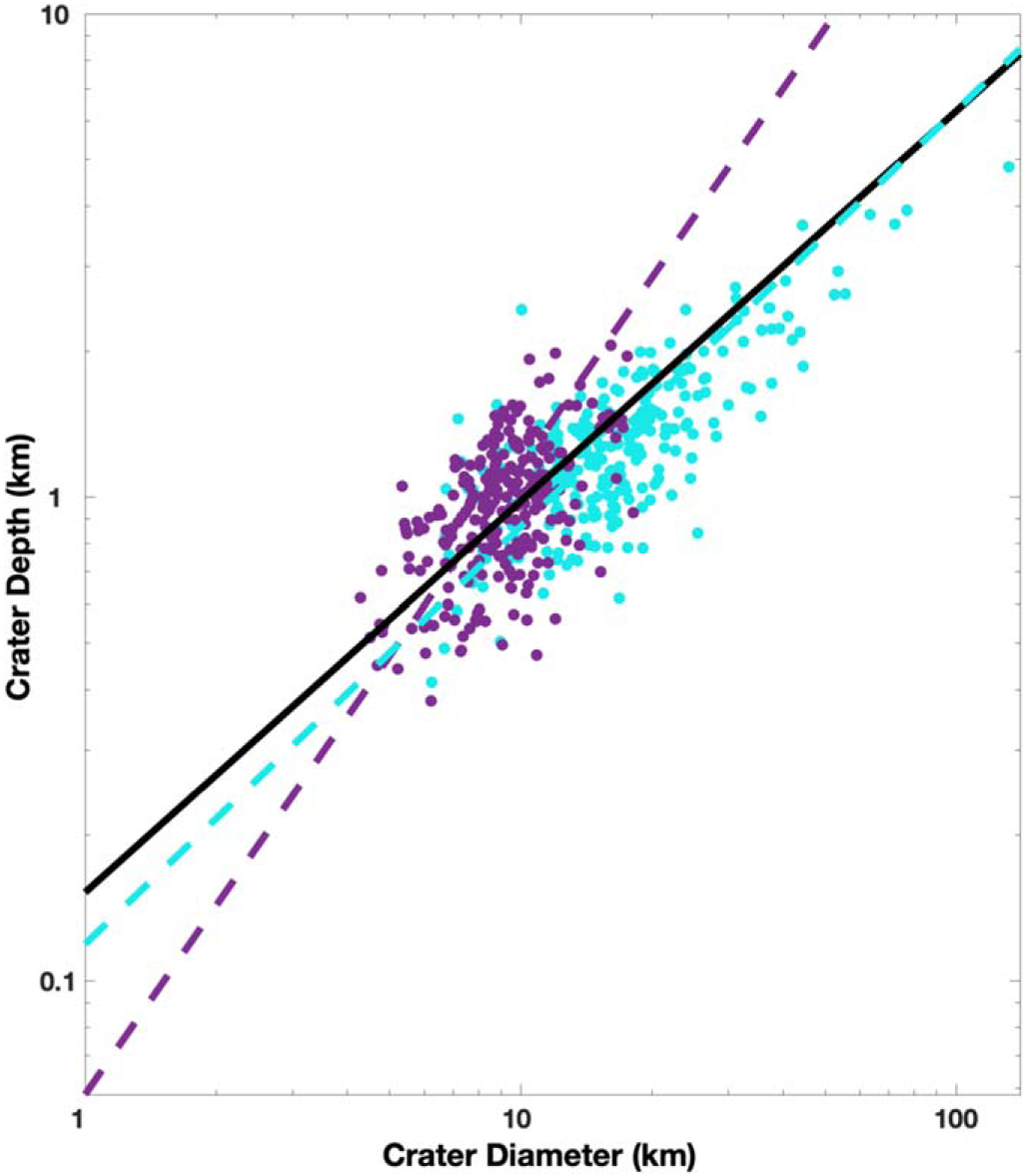
Crater depth as a function of crater diameter in log space. The black solid line is the linear fit to all the data, while the purple and cyan dashed lines correspond to the simple and complex craters, respectively.

**Figure 9.**
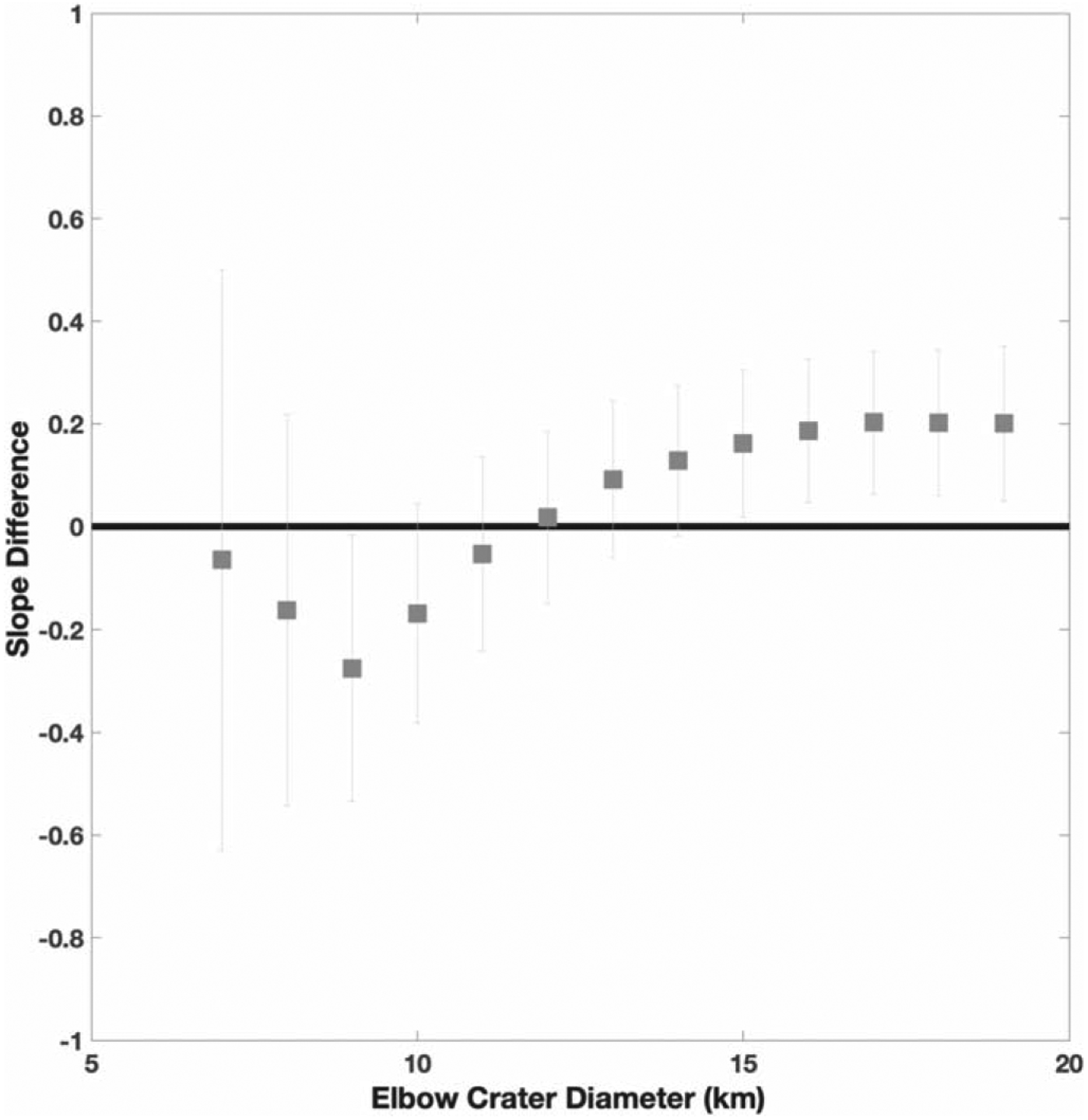
The difference in the LSF slopes as a function of the crater diameter considered the elbow location (i.e., below and above this value separate LSFs are fit to the data). The error bars are the propagated uncertainties in the slope fits. The black solid line notes where the difference is indistinguishable from zero (i.e., the two lines may be the same).

A more often used method involves analysis of crater depth as a function of diameter, which typically follows a power law of the form *d* = *bD*^*β*^ such that in log space log_10_(*d*) = *β* log_10_(*D*) + log_10_(*b*). In Figure 8, we fit an LSF, following the York method, to the simple and complex craters. The LSF slope is 0.81 ± 0.26 for all craters, 1.3 ± 0.83 for simple craters, and 0.86 ± 0.41 for complex craters to a 95% confidence. Our values are within error similar to those derived by White et al. (2013). The intercept of the two best-fit power laws was then considered the transition diameter. The intercept occurs at 5.2 ± 4.5 km. Following a similar process, another method uses the same morphometric information but is agnostic to the crater morphology and instead seeks where a change in slope exists in the data set. To do this, we stepped through crater diameters in small incremental steps of 1 km and fit an LSF on either side. We then found the difference of the LSF slopes and corresponding error. The point where the differences in slope are distinguishable from zero is considered where an “elbow” exists in the data set because the slopes would be statistically distinguishable. We plot these differences in Figure 9, which shows that the elbow may lie between 9 and 15 km.

**Figure 10.**
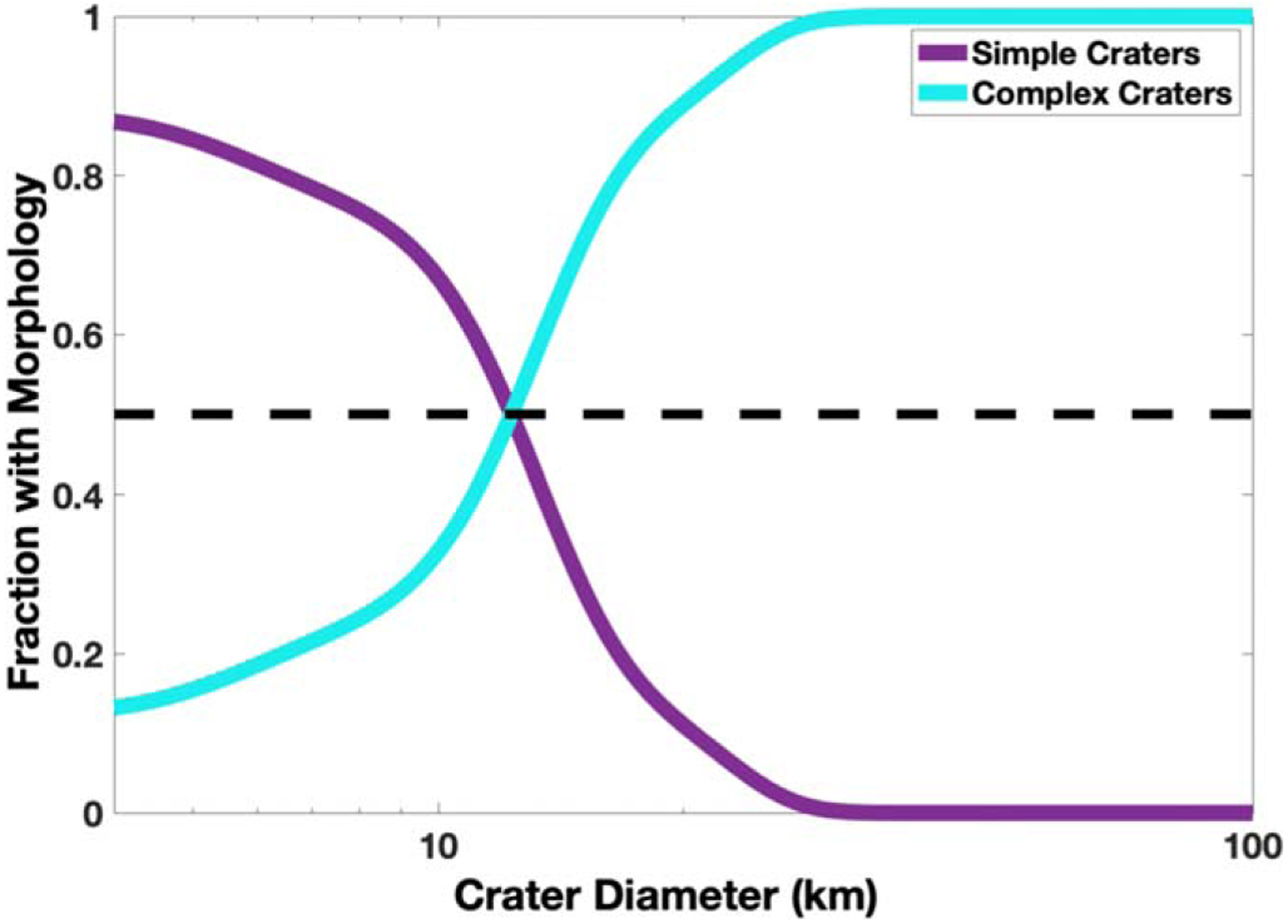
Distribution plot of the fraction of craters that are simple (purple) or complex (cyan) morphologies. The black dashed line denotes where the fraction is 0.5, which is used to estimate the simple-to-complex crater transition diameter. The fraction of simple craters does not reach 1 here since the minimum crater diameter used in this study was 4.3 km.

Finally, we created a probability distribution function (pdf) for the studied crater population, as well as for the identified simple and complex craters following the method described in Robbins et al. (2020). In this technique, every measured crater diameter is represented by a Gaussian distribution with a mean value of *D* and a standard deviation given as 0.25*D*. The Gaussians are then summed for the entire crater population, as well as for only simple and complex craters to create a pdf for each case. The ratio of the simple pdf to the population pdf and that of the complex pdf to the population pdf are shown in Figure 10. The point where these ratioed pdf’s suggest that 50% of craters are simple (or complex) is then used to approximate *D*_*t*_. This results in **$D_{t}=12.3_{-0.6}^{+1.6}$** km, where the error is estimated by varying the standard deviation in the Gaussian distribution by 2.

**Figure 11.**
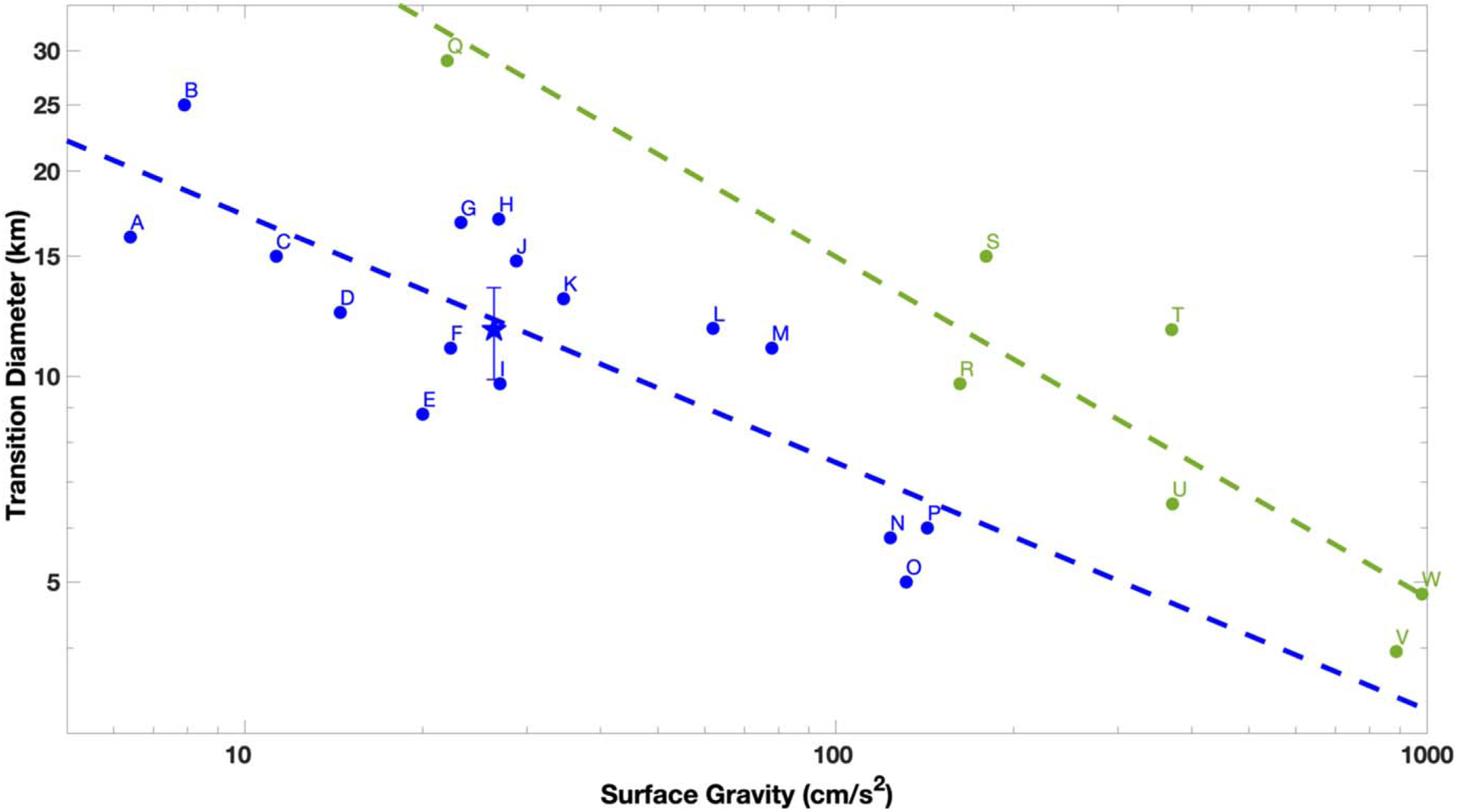
Simple-to-complex crater transition diameter as a function of surface gravity for both icy (blue) and rocky (green) bodies. The dashed line is the best-fit power law to the transition values. Our new transition diameter for Rhea of 12 ± 2 km is denoted as a star with corresponding error bars. The labels represent (A) Mimas (Schenk 1989), (B) Miranda (Schenk 1989), (C) Enceladus (Schenk 1991), (D) Tethys (White et al. 2017), (E) Umbriel (Schenk 1989), (F) Iapetus (Giese et al. 2008), (G) Dione (White et al. 2017), (H) Ariel (Schenk 1989), (I) Ceres (Hiesinger et al. 2016), (J) Charon (Robbins et al. 2020), (K) Oberon (Schenk 1989), (L) Pluto (Robbins et al. 2020), (M) Triton (Schenk 1989), (N) Callisto (Schenk 2002), (O) Europa (Moore et al. 2001), (P) Ganymede (Schenk 1991), (Q) Vesta (Vincent et al. 2014), (R) Moon (Pike 1988), (S) Io (Zahnle et al. 1998), (T) Mercury (Susorney et al. 2016), (U) Mars (Robbins & Hynek 2012), (V) Venus (Schenk 2002), and (W) Earth (Pike 1980).

Combined, the four methods to estimate *D*_*t*_ suggest a weighted average of 12 ± 2 km. The methods also agree well with each other. Considering gravity scaling laws, Iapetus (0.22 m s^−2^), Charon (0.29 m s^−2^), and Rhea (0.26 m s^−2^) should have similar *D*_*t*_ if their surfaces also have similar properties. In their recent work, Robbins et al. (2020) found a *D*_*t*_ for Charon of ~15 km. Giese et al. (2008) found a *D*_*t*_ of 11 ± 3 km for Iapetus. Previously, White et al. 2013 found a value of 4.7 km for Rhea, while Chapman & McKinnon (1986) and Schenk (1991) found 15 and 12.4 km, respectively. Our newly derived value for Rhea agrees well with Charon and Iapetus; thus, these bodies may have similar surface properties. We note that White et al. (2013) derived *D*_*t*_ by finding the intersection of the power-law fits to their simple and complex crater depth and diameter data. In their work, only simple craters with fresh morphologies were included. Our derived value using the same technique, except for craters of all degradation states and using the York method to derive an LSF in order to fully account for the measurement uncertainties, was 5.2 ± 4.5 km, which is within error similar. This may indicate that this technique can only provide a lower bound estimate. In Figure 11, we summarize a collection of reported *D*_*t*_ for both icy and rocky surfaces, along with our newly derived *D*_*t*_ for Rhea. The trend of transient crater diameters as a function of gravity (*g*) is well described by a power law, where for rocky bodies the best fit follows *D*_*t*_ = (149.2 ± 3.1)*g*^−0.5±0.2^ and for icy bodies the best fit follows *D*_*t*_ = (39.7 ± 1.7)*g*^−0.4±0.1^. Our new Rhea value fits well within the expected range for icy, low-gravity surfaces. Interestingly, the simple-to-complex crater transition diameter on the main belt asteroid Ceres, where gravity is *g* = 0.27 m s^−2^, occurs between 7.5 and 12 km (Hiesinger et al. 2016). In contrast, Vincent et al. (2014) report that the transition from simple to complex craters starts at 38 km on the asteroid Vesta (*g* = 0.22 m s^−2^). As such, Ceres is more similar to icy than rocky worlds. Indeed, Schenk et al. (2021) found that crater morphologies on Ceres are similar to those found on the icy moons of Jupiter and Saturn, indicating that the Cerean crust may be dominated by ice.

Besides the transition from simple to complex craters, Schenk (2002) found that on Callisto, Ganymede, and Europa an additional change in overall crater morphometry occurs whereby large complex craters begin to have shallower depths than smaller complex craters. This transition occurs at *D* ≈ 26 km for Callisto and Ganymede and *D* ≈ 8 km for Europa, after which crater depths are shallower than would be predicted by a best-fit power law (Schenk 2002). These changes are also accompanied by differences in crater morphology, from central peak craters to central pit and dome craters. Such a change in overall crater morphometry with increasing complex crater diameter may be the result of the crater excavation process sampling a weaker, warm ductile ice layer at depth. Differences between the satellites would be a result of differences in internal structure. Indeed, Ganymede and Europa are fully differentiated (Anderson et al. 1996, 1998), while Callisto may only be partially differentiated (Anderson et al. 2001). Here we tested for this additional transition by finding the fraction of craters above and below the best-fit power law for complex craters in Figure 8. Craters with *D* > 40 km are in general shallower than the best-fit power law. This may imply that the transition to warm ductile ice on Rhea occurs deeper than on Callisto, Ganymede, and Europa. However, there are only 13 craters within our data set with *D* > 40 km, so such an interpretation could be biased and is not robust. Rhea’s gravity field suggests an undifferentiated body, though a partially differentiated interior cannot be ruled out (Tortora et al. 2016). Indeed, coupled geophysical and orbital evolution models of the Saturn system would suggest that Rhea may have been able to retain an ocean for 1.5 Gyr after its formation (Neveu & Rhoden 2019). A lack of a clear transition for complex craters to shallower depths on Rhea may indicate that at least the majority of craters in our study area formed when such a rheological change in ice at depth was either nonexistent, which would support an undifferentiated body, or deeper than sampled by the craters, which could support a partially differentiated interior.

## 4. Conclusion

We used a high-resolution, regional digital elevation map to conduct a morphometric study of a sample of craters on Rhea, particularly to investigate the dominant post-impact modification process. Our new morphometric measurements agree well with previous results for crater sizes, depths, and wall slopes (Schenk 1991; Rivera-Valentín et al. 2012; White et al. 2013). Overall, crater wall slopes are on average 12°, ranging from 4° to 26°. Maximum crater wall slopes are near the angle of repose for rounded grains, which is 25° (Kleinhans et al. 2011). Interestingly, the range of measured crater wall slopes is similar to the slopes reported for Iapetus’ equatorial ridge (Lopez Garcia et al. 2014). Overall, we found an average depth-to-diameter (*d*/*D*) ratio of 0.1 for a range of crater degradation states, which is more similar to asteroidal values (Sullivan et al. 1996; Robinson et al. 2002; Burchell & Johnson 2005) than lunar values (e.g., Pike 1974).

Depth-to-diameter ratio has previously been used to infer relaxation due to endogenic heating, which would also shallow the crater wall slopes. Thus, here we studied the relationship between crater wall slopes and *d*/*D*. We found that crater wall slope is a linear function of *d*/*D*. This suggests that most craters in our study area are modified by a process that nearly equally alters the *d*/*D* and crater wall slope. This is most consistent with past heating events as the dominant post-impact modification process on Rhea as previously suggested (Schenk et al. 2011; White et al. 2013). However, we also identified two groups that are not well predicted by this linear model. The first group is characterized by steeper crater wall slopes (>15°) and is dominated by complex craters, and the second group by shallower crater wall slopes (<15°) and is dominated by simple craters. Other than crater wall slope and overall morphology, though, these two groups of craters did not exhibit other properties, such as clustering, as would be expected for secondaries. As such they may represent part of the evolution of crater morphologies on Rhea.

We also worked to derive the simple-to-complex crater transition diameter, which is largely dependent on gravity and the body’s surface material properties. Because there is no best practice as of yet on analytical techniques to derive this property (see Robbins et al. 2020 for details), we employed four different methods. Together, we obtained a weighted average of 12 ± 2 km. Our derived value is consistent with bodies of similar gravities, such as Charon (Robbins et al. 2020), Iapetus (Giese et al. 2008), and, interestingly, Ceres (Hiesinger et al. 2016). The similarity of simple-to-complex crater transition diameter between the comparably sized icy bodies may suggest similar surface properties. Additionally, large complex craters in our study area do not clearly exhibit a depth shallowing at larger sizes. This is in contrast to Europa, Ganymede, and Callisto, where an additional morphology transition occurs for complex craters due to differences in interior properties. This may lend support to the interpretation from gravity data that Rhea is likely not fully differentiated (Tortora et al. 2016). Further analysis of other bodies in light of high-resolution data and advanced statistical analysis is warranted to provide a consistent technique that allows for interplanetary comparisons.

## Figures and Tables

**Figure 12.**
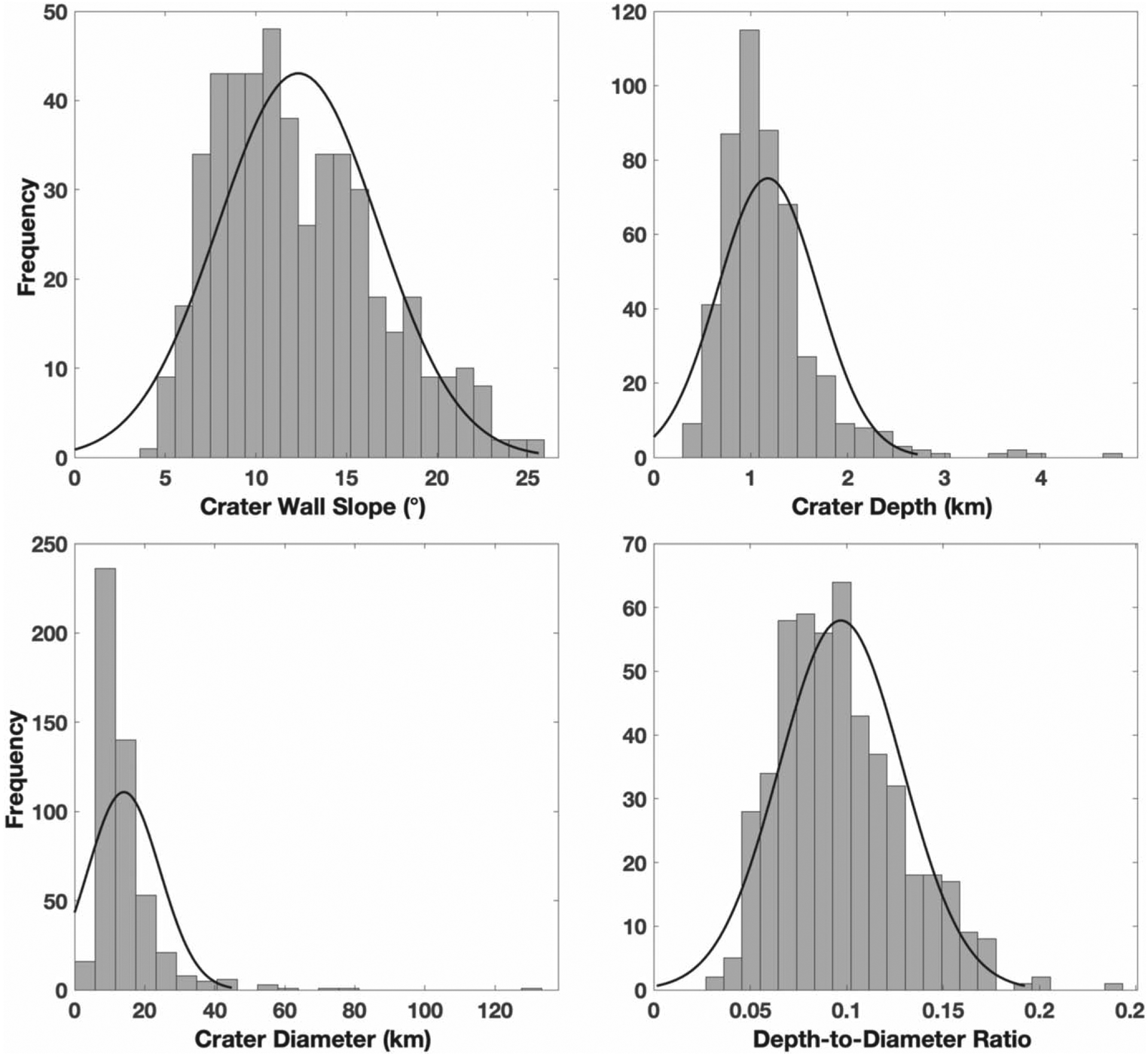
Histogram of the data collected for crater (A) wall slope, (B) depth, (C) diameter, and (D) calculated depth-to-diameter ratio compared to a Gaussian distribution fit of the data (black line).

**Figure 13.**
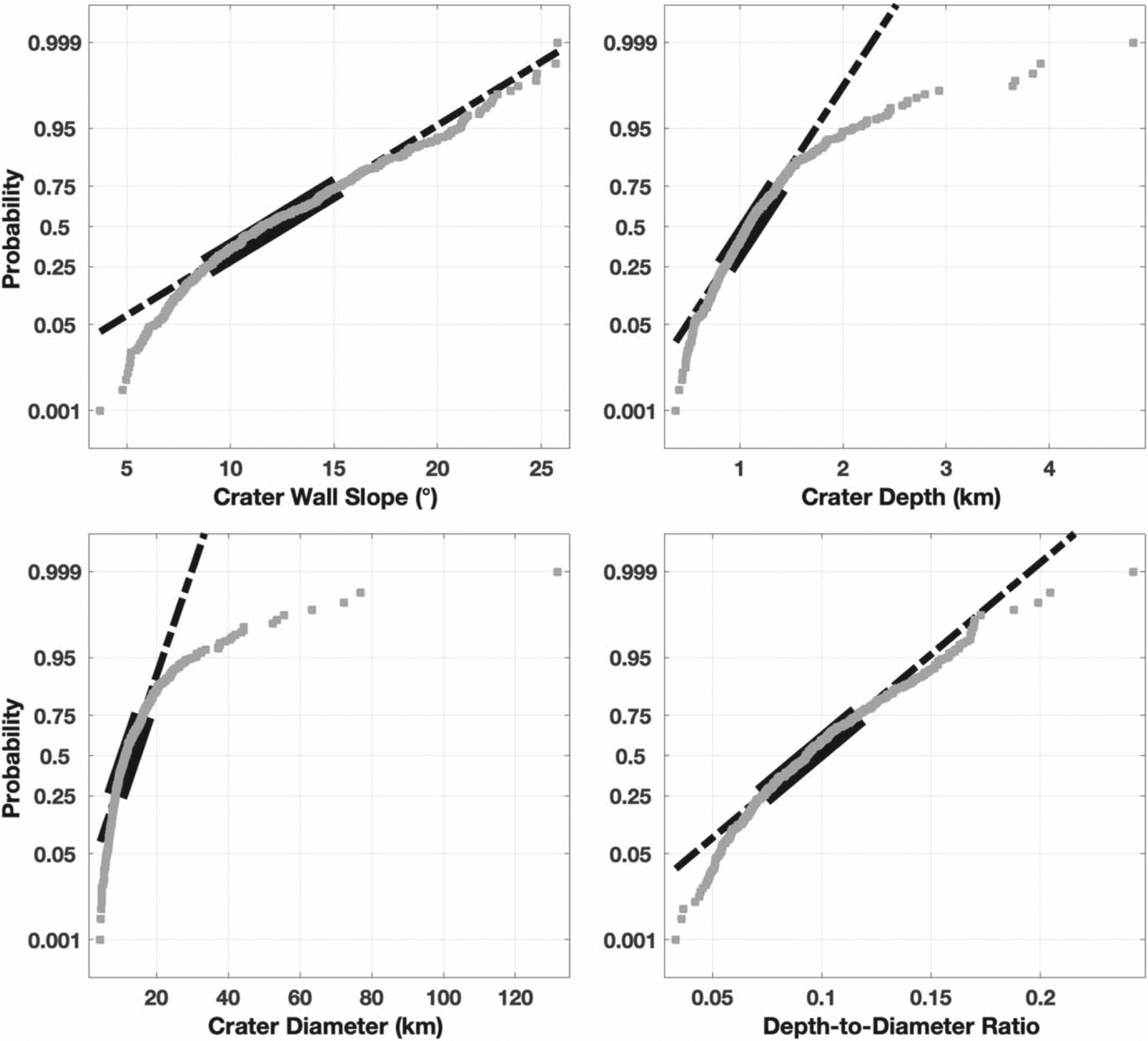
Normal probability plot for crater (A) wall slope, (B) depth, (C) diameter, and (D) calculated depth-to-diameter ratio. Gray symbols are the empirical probability versus the data value for each point in our data set. The dashed black line is a linear fit, the portion of the linear fit that corresponds to the 25th and 75th percentiles is shown as a thick, solid line.

**Table 1:** Summary of the Collected Crater Morphometry Measurements

Classification	Count	Parameter	Diameter (km)	Depth (km)	*d/D*	Crater Wall Slope (deg)
Simple	248	Max	18.1	2.1	0.2	24.8
		Min	4.3	0.4	0.05	5.2
		Median	9.0	1.0	0.11	13.1
		Mean	9.3 ± 2.6	1.0 ± 0.3	0.11 ± 0.03	13.4 ± 4.4
Complex	261	Max	131.9	4.8	0.24	25.8
		Min	6.2	0.4	0.03	4.0
		Median	16.1	1.2	0.08	10.4
		Mean	18.9 ± 12.4	1.4 ± 0.6	0.08 ± 0.03	11.2 ± 4.2
All	509	Max	131.9	4.8	0.24	25.8
		Min	4.3	0.4	0.03	4.0
		Median	11.5	1.1	0.09	11.5
		Mean	14.2 ± 10.3	1.2 ± 0.5	0.10 ± 0.03	12.3 ± 4.4

## Appendix

The confidence testing applied in this work relies on the assumption that the distribution of the parameters studied follow a Gaussian distribution. Here we validate that assumption by conducting normality tests for the data collected for crater diameter, depth, and crater wall slope, as well as the calculated depth-to-diameter ratio. The normality of each parameter was evaluated in two ways. First, in Figure 12, we compare a histogram of the data to a Gaussian distribution with the same mean and standard deviation of the data. Crater wall slope and depth-to-diameter ratio are well modeled with a Gaussian distribution. Crater depth is generally well modeled by a Gaussian, but has a long tail. On the other hand, crater diameter is not well modeled as it is both skewed and has a long tail. The second test employs a normal probability plot, where the data are plotted against its empirical probability. Normality can be inferred if the data generally follow a linear trend. As seen in Figure 13, all of the measured parameters follow closely a linear trend between the 25th and 75th percentiles. Generally, it is reasonable to assume a normal distribution for crater wall slope and depth-to-diameter ratio. Measurements of crater depth significantly deviate from a linear trend for depths larger than 2 km, which is some 5% of the data. Measurements of crater diameter significantly deviate from a linear trend for craters larger than some 30 km, which is some 5% of the data. Together these tests suggest that measurements of crater diameter are likely not normally distributed. However, our conclusions primarily focused on the analysis of crater wall slope and depth-to-diameter ratio (i.e., Figure 5), which are reasonably normally distributed, and thus the applied confidence testing was appropriate. Confidence testing for analysis on crater depth and diameter (i.e., Figure 8) may not be as robust; however, in Section 3 we employed various methods besides confidence testing that were all reasonably in agreement.
